# Formation and Characterization of the Recast Layer Formed on Inconel 718 during Wire Electro Discharge Machining

**DOI:** 10.3390/ma16030930

**Published:** 2023-01-18

**Authors:** Bandar Alkahlan, Thamer Tabbakh, Abdulaziz Kurdi, Alokesh Pramanik, Animesh K. Basak

**Affiliations:** 1 Advanced Materials Institute, King Abdulaziz City for Science and Technology, P.O. Box 6086, Riyadh 11442, Saudi Arabia; 2 Microelectronics and Semiconductors Institute, King Abdulaziz City for Science and Technology, P.O. Box 6086, Riyadh 11442, Saudi Arabia; 3 School of Civil and Mechanical Engineering, Curtin University, Bentley, WA 6102, Australia; 4 Adelaide Microscopy, The University of Adelaide, Adelaide, SA 5005, Australia

**Keywords:** wire EDM, Inconel, recast layer, micro-pillar compression, EBSD, microstructure

## Abstract

The present work investigates the formation and microstructural and micro-mechanical characterization of the recast layer that formed on Inconel 718 alloy in the course of the wire electro-discharge machining (WEDM). The as-machined surface contains globules, shallow cracks, and re-deposition of molten materials, together with the elements from the decomposition of wire electrode and electrolyte, which does not exceed beyond the surface of the recast layer. Under presently investigated machining parameters, the recast layer was about 6.2 ± 2.1 µm thick. There was no presence of a heat-affected zone (HAZ), as otherwise indicated for other hard-to-cut materials. The transmission electron microscopy (TEM) and electron back-scattered diffraction (EBSD) investigations show that the microstructure of the recast layer is similar to that of bulk alloy. Micro-mechanical characterizations of the recast layer were investigated via in-situ micro-pillar compression on the micro-pillars fabricated on the recast layer. The strength of the superficial layer (1151.6 ± 51.1 MPa) was about 2.2 times higher than that of the base material (523.2 ± 22.1 MPa), as revealed by the in-situ micro-pillar compression.

## 1. Introduction

Wire electro-discharge machining (WEDM) is a versatile machining technique regularly employed to shape/fabricate intricate features in hard-to-cut materials, like Ti and Ni-based alloys, metal matrix composites, and high-strength steel [1]. WEDM, in general, has a number of advantages over other machining processes during the fabrication of such hard and difficult-to-machine materials. However, such advantages do not come unconditionally, as the machined surface after WEDM is usually rough and contains numerous cracks, pores, re-solidified materials, elements of electrode material, and oxidation products that took place due to electrolysis of the electrolyte [2]. During the WEDM process, a fraction of molten material is flushed away due to applied flushing pressure, whereas the remaining fraction gets re-solidified on the workpiece surface to form a superficial layer, often termed recast layer in the literature [3]. The extent of this layer depends on the machining parameters used during the process [2]. For example, as reported by Pramanik et al. [2], in the case of the WEDM of Ti6Al4V alloy, a 10 µs of pulse on time, 15 MPa of flushing pressure, and 1400 gf wire tension induce about 10 µm thick recast layer. In contrast, a 4 µs of pulse on time, 15 MPa of flushing pressure and 1400 gf wire tension induce about 4 µm thick recast layer. Recast layer thickness primarily increases with the increase of spark energy, discharge current, and duration of current pulse [2]. Prior to application, such WEDM parts usually go through surface modification, such as etching, grinding, polishing, particle blasting, and/or milling to remove such recast layer (surface defects) [4]. Towards that, a good understanding of the microstructure and mechanical properties of such recast layer is necessary. There is only a handful of reports available in the literature that mainly state the presence of the recast layer on WEDM processed Inconel 718 in terms of machining parameters, without any details of microstructure and mechanical properties [1,3,5,6]. The main reason behind such limited available information on the recast layer of Inconel 718 alloy is the lack of experimental setup, as discussed below.

The flow behavior (strength) of a given material can be found from the tensile testing of the materials in the form of stress-strain curves. This technique is mostly limited to bulk materials toward the fabrication of common ‘dog-bone’ type samples. Due to the given profile of the recast layer, this traditional tensile testing cannot be employed on that. However, this limitation can be overcome by employing micro-pillar compression, which was successfully applied in different coating systems [7,8,9] as well as in bulk materials [10,11], as reported in the literature. The load-displacement curves obtained from such in-situ micro-pillar compression can be converted into strain-stress curves that provide valuable information regarding a fundamental understanding of the deformation of materials. Besides that, quantitative mechanical properties of the investigated materials can also be obtained.

Towards that, the present work investigates the microstructural characteristics along with micro-mechanical properties of the recast layer that forms on Inconel 718 during WEDM. The outcome of the present research will provide a deep understanding of the nature of the recast layer.

## 2. Materials and Methods

### 2.1. WEDM of the Inconel 718

**Figure 1:**
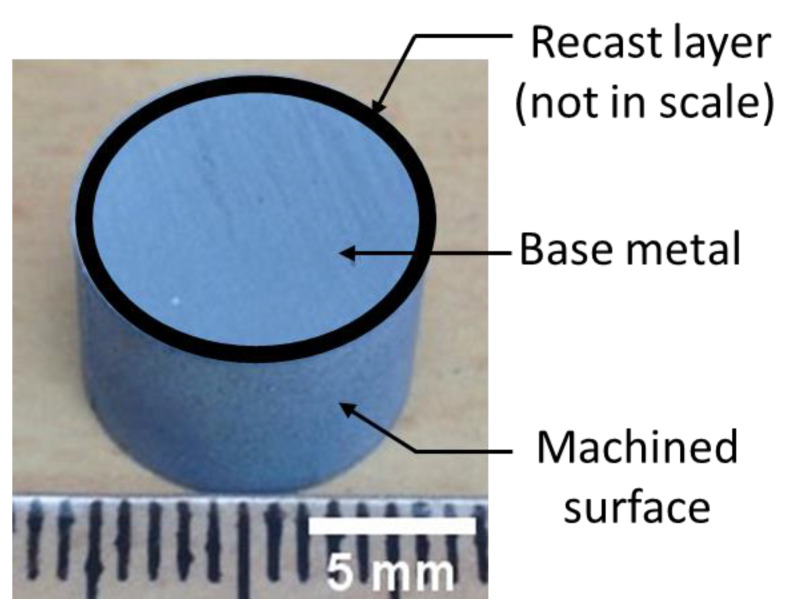
Optical photo of the cylindrical block cut by WEDM from the workpiece with the location of the recast layer (overlay) and machined surface.

The Inconel 718 alloy was commercially procured from Rolled Alloys Ltd. (Singapore) in the form of a round bar with following nominal composition (wt.%) as provided by the supplier: 52.5 Ni-19 Cr-3.0 Mo-5.1 Nb-0.90 Ti-0.50 Al-18 Fe. According to the supplier, the alloy was first melted in vacuum induction furnace (968 °C), followed by forgings to give the bar shape, and then subjected to solution seat treatment to make it precipitation harden. The alloy was subjected to WEDM with the help FANUC ROBOCUT α 0iD wire-EDM machine. At first, a rectangular block of 70 × 50 × 10 mm was cut from an Inconel 718 alloy slab. Then, WEDM was used to cut cylinders of 12 mm diameter with 10 mm height, as shown in Figure 1, from that rectangular block.

A Ø 250 µm brass wire (coated with zinc) was used as a tool electrode. The following input parameters were used during the WEDM process: Spark cycle 16 µs, open voltage time 70 µs, voltage off time 10 µs, power 35 W, wire tension 2500 gf, dielectric flow rate 8 L/min, and wire speed 260 mm/min. Deionized water was used as dielectric medium. These parameters were selected based on the recommendation by Newton et al. [5] and Nair et al. [12]. 

### 2.2. Microstructural Characterization of the WEDMed Surface

After WEDM, morphology of the machined surfaces, as well as the cross-sectional view, were inspected by field emission scanning electron microscopy (FESEM, Quanta 450, FEI, Lausanne, Switzerland) coupled with Oxford Instruments^®^ energy dispersive X-ray spectroscopy (EDX, Abingdon, UK). The cross-section of the sample was prepared by resin embedding followed by metallographic polishing in Struers automatic metallographic polisher with final polishing in colloidal silica suspension. Transmission electron microscopy (TEM) foils on the recast layer were fabricated by the focused ion beam (FIB-SEM, Helios Nova Nanolab 600, FEI: Lausanne, Switzerland) with final polishing at 93 pA current at 30 kV. TEM investigation was carried out on probe-corrected Titan Themis (FEI) TEM at 200 kV. 

### 2.3. Fabrication of the Micro-Pillars for In-Situ Compression

**Figure 2:**
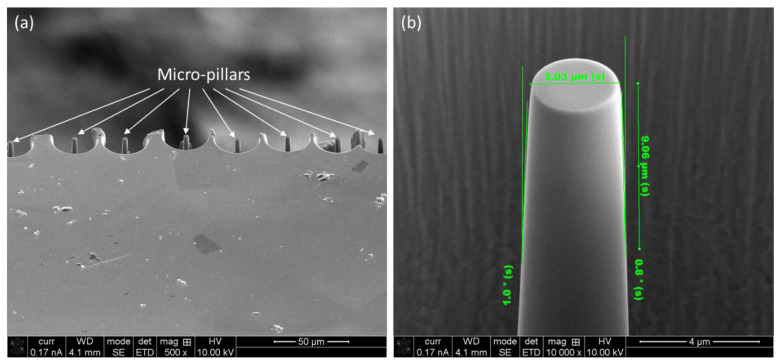
(**a**) A series of FIB-SEM prepared micro-pillars in the middle of Ø 30 μm pit on the recast layer, and (**b**) high magnification images on one of the micro-pillars together with dimensions.

The compressive strength of the recast layer, as well as of bulk monolithic materials, was investigated via in-situ micropillar compression in the Hyistron PI-88 (Hyistron, Australia) system. Towards that, a 3 μm diameter pillar was fabricated by FIB-SEM. The compression tests were carried out in displacement control mode using a 3 nm/s loading rate. To avoid any artefact in experimental results, the micro-pillars were fabricated in the middle of a 30 μm diameter pit so that the indenter would not touch the surrounding sample surface except the micro-pillar. At first, the milling towards micro-pillar compression was conducted at a relatively higher current (9.3 nA current at 30 kV) followed a progressively lower current with final polishing at 0.28 nA at 30 kV to obtain a smooth pillar surface. The obtained load–displacement graphs during micro-pillar compression were converted to stress–strain curves. For this, the method reported by Misra et al. [13] was employed. This method takes into consideration the taper profile of the micro-pillars with adequate corrections on that. Successful implementation of this method was reported by Kurdi et al. [8] on in situ compression of Co/Sn multi-layered coatings and Basak et al. [11] on SiC-reinforced metal matrix composites (MMCs). At least seven different compression tests were carried out in each case for statistical analysis, and three of them in each case were reported for a better presentation of the graphs. A representative SEM micrograph of a series of micro-pillars made on the recast layer is shown in Figure 2a, together with a higher magnification image in Figure 2b. As evident from Figure 2b, the micro-pillars are slightly taper (<2°), which is unavoidable due to the interaction of the ion beam with the materials [14]. The final diameter of the pillars was 3 µm with 9 µm height, which was equivalent to a 1:3 ratio, to avoid buckling during compression [15].

## 3. Results and Discussion

### 3.1. Physical Characterization of the Recast Layer

#### 3.1.1. SEM Characterization

**Figure 3:**
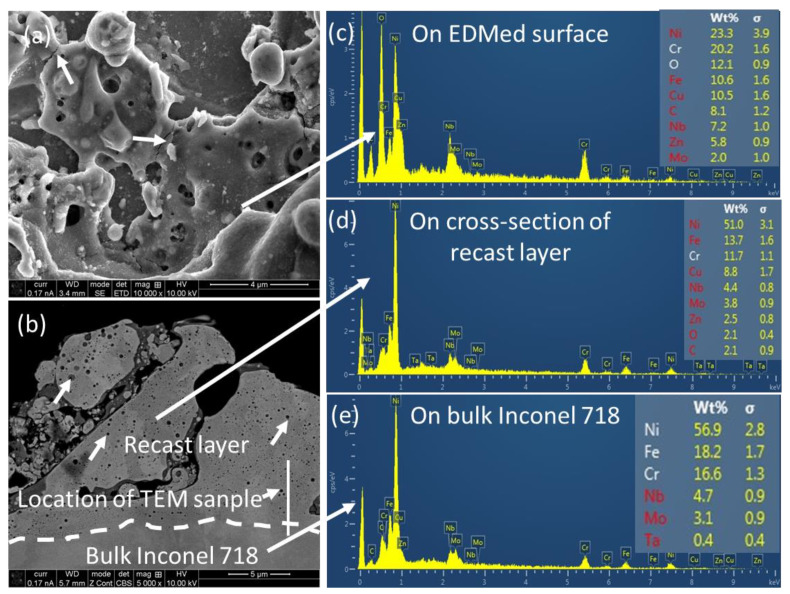
Representative features on the machined surface of Inconel 718 after WEDM on (**a**) cylindrical surface and (**b**) cross-together with energy-dispersive X-ray spectroscopy (EDX) spectra on (**c**) cylindrical surface, (**d**) cross-section, and (**e**) bulk material.

After the WEDM process, morphology of the machined surface of the cylindrical block is shown in Figure 3a. It is evident that the machined surface (i.e., recast layer) contains a number of features, such as molten droplets, depressions, shallow cracks, craters of different sizes, and re-solidified debris (marked with arrows in Figure 3a). These features form due to the melting of the workpiece materials as well as tool electrode during the WEDM process, followed by quick re-solidification of molten materials due to rapid cooling in the WEDM zone. This rapid cooling causes the molten/vaporized material to be vitrified together with generation of residual stresses. As stress build-up continues, it eventually exceeds the ultimate strength of the material at some point and releases the stress in the form of cracks on EDM of Ti-alloy, as explained by Hasçalık et al. [16]. The cross-sectional SEM image of the recast layer is shown in Figure 3b, where the presence of molten droplets and re-solidified debris is evident and quite distinct from that of bulk material, which is free from such features. The average thickness of the recast layer was about 6.2 ± 2.1 µm. Similar features of the recast layer that form on Ti6Al4V alloy on the course of WEDM was reported in previous communication [17]. However, it is interesting to note that there was no evidence of heat affected zone (HAZ), which was otherwise reported in the case of WEDM of Ti6Al4V alloy [17]. The reason behind that was the relatively higher thermal conductivity of Inconel 718 (11.2 W m^−1^ K^−1^) [5] than that of Ti6Al4V (6.7 W m^−1^ K^−1^) [17]. Due to this, the heat can dissipate quickly in the surrounding without the formation of HAZ. The EDS spectrum on the recast layer, both in surface (Figure 3c) and cross-section (Figure 3d), displays the existence of the Cu and Zn peaks which came from the working electrode, along with base alloy (Ni, Fe, Cr, Nb, Mo, and Ti). Furthermore, C and O peaks were also present, most probably as a result of dielectric decomposition due to electrolysis, as reported by Pramanik et al. [2]. These peaks were not evident in the bulk materials (Figure 3e). This means that the accumulation of elements, like Cu, Zn, C, and O, is confined only within the surface and does not diffuse into the bulk alloy due to a high cooling rate.

#### 3.1.2. TEM Characterization

**Figure 4:**
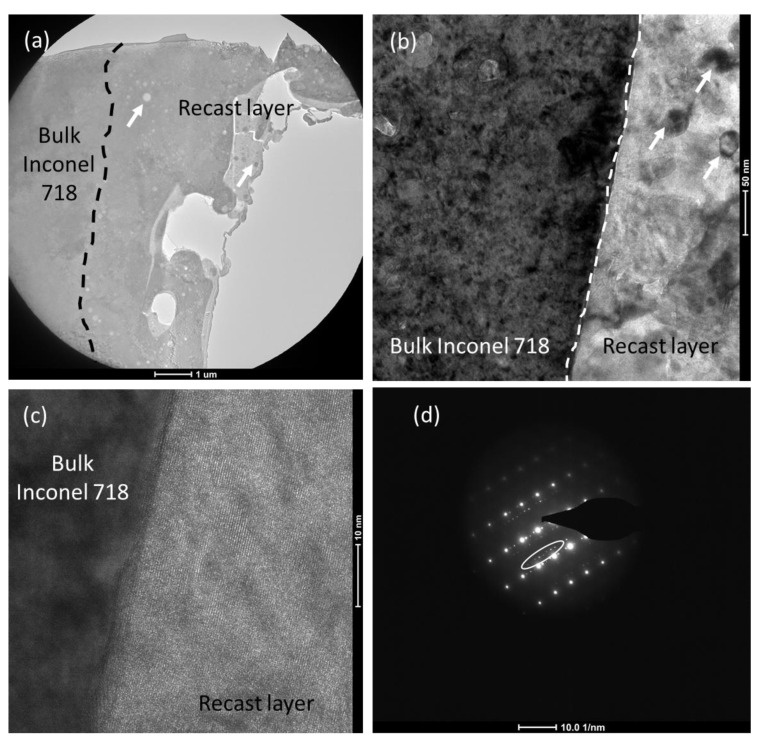
Representative transmission electron microscopy (TEM) images of the recast layer: (**a**) Brightfield (BF)-TEM image together with bulk material, (**b**) the interface of the recast layer with the bulk material, (**c**) high-resolution (HR) TEM image of the interface, and (**d**) corresponding selected area electron diffraction (SAED) pattern.

The morphology of the recast layer was further analyzed by TEM, and the TEM sample was prepared at a location marked with a white bar in Figure 3b, which contains both bulk material and the recast layer. Representative TEM images are shown in Figure 4, where Figure 4a shows the bright field TEM (BF-TEM) image of the recast later together with bulk material. The morphology of the recast layer is distinct from that of bulk material that contains cracks, molten droplets, and globules, as marked with arrows. As the recast layer formed all around the cylindrical block, the images in Figure 4 are representative and independent of the location from where the TEM foil was made on the cylindrical block. Figure 4b shows the interface of the recast layer with the bulk material. Whereas the bulk material represents the typical microstructure of Inconel 718 [18], the recast layer contains second-phase particles/derbies that most probably results from the solidification of the molten materials. The existence of these phases in the recast layer was reported in the literature [6,16] via X-ray diffraction and for the first time, evidenced via TEM investigation. A high-resolution TEM (HRTEM) image of the interface (Figure 4c) confirms the metallurgical bonding of the recast later with the bulk materials without any micro-void formation [11]. Figure 4d shows the selected area electron diffraction (SAED) pattern with the presence of secondary diffraction points from the second phase/debris, as marked with arrows. The SAED pattern also confirms the face-centered cubic (FCC) solid solution of Inconel, i.e., austenite (γ) [18]. There was a change in lattice orientation along the interface and the SAED pattern, confirming the cubic orientation (FCC and BCT/TCP) of the crystal structure of the recast layer.

#### 3.1.3. Electron Backscattered Diffraction (EBSD) Characterization

**Figure 5:**
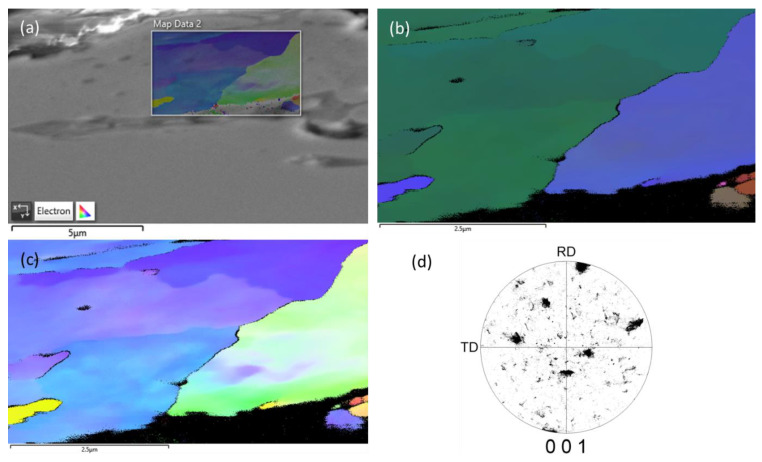
EBSD investigation of the recast layer: (**a**) Electron image with overlay map area, (**b**) image quality (IQ) map, (**c**) inverse pole figure (IPF) map, and (**d**) pole figure (PF) map.

To acquire a better understanding of the texture orientation of the recast layer, electron backscattered diffraction (EBSD) analysis was carried out, as shown in Figure 5. Figure 5 shows an electron image with overlay map area (Figure 5a), image quality (IQ) map (Figure 5b), inverse pole figures (IPF) map (Figure 5c), and pole figure (PF) map (Figure 5d). As can be seen from the EBSD investigation, there was no characteristic texture of the recast layer. It is important to note that, due to the limited profile of the recast layer, it was not possible to analyze grain size distribution in the recast layer.

### 3.2. Micro-Mechanical Characterization of the Recast Layer

**Figure 6:**
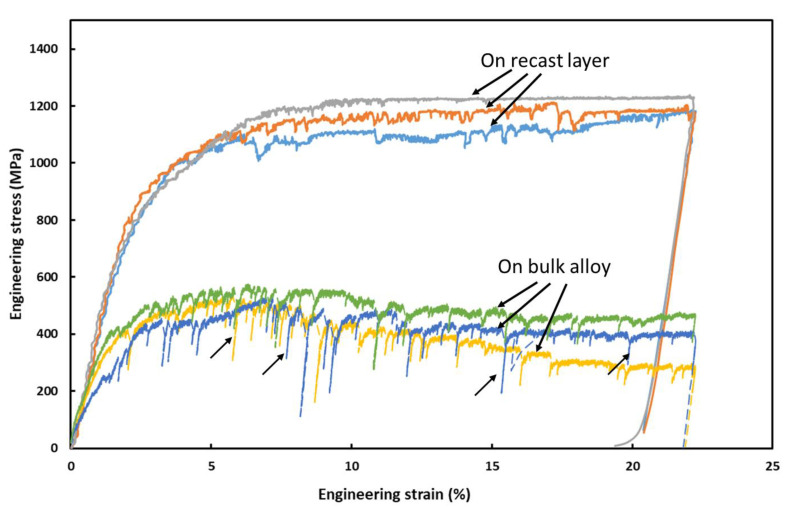
Representative stress–strain curves on recast layer and bulk Inconel 718 alloy derived from load-displacement curves under in-situ micro-pillar compression.

The typical stress-strain behavior of the recast layer, along with bulk material under in-situ micro-pillar compression, is shown in Figure 6. The occurrence of the elastic and plastic flow of the material was evident as the deformation of the material continued under compression. Moreover, the stress–strain behavior of the recast layer and bulk material was comparable in nature except for the magnitude of stress. It was obvious from Figure 6 that stress accommodation of the recast layer is much higher than that of bulk materials. The strength and elastic modulus of the recast layer, as well as bulk material, were calculated [13,19] from such curves, as reported in Table 1. The recast layer possessed higher strength (1151.6 ± 51.1 MPa) as compared to the bulk material (523.2 ± 22.3 MPa). The surge in strength of the superficial layer was due to the presence of various carbides and oxide [16] particles in that layer, which contribute to the strength by blocking dislocation/movement of material as second-phase particles [5]. The sudden drops in the stress–strain curve are a typical characteristic of micro-pillar compression, which was related to the existence of the slip/shear band formation that acted as a major load-bearing mechanism. This behavior was also known as serrations or jerky flow as dislocation starved and represents the annihilation of dislocations and their regeneration. Once the applied load exceeds the critically resolved shear stress [20], the crystal structure cannot rearrange anymore, and the formation of micro-voids takes place, followed by the plastic flow of the materials along favored slip/shear planes. Similar behavior of stress-strain curves on the recast layer that formed on Ti6Al4V alloy subjected to the WEDM process was reported by Basak et al. [21,22]. However, the main contrast was, the recast layer formed on Ti6Al4V was not able to sustain considerable strain accommodation (<3% [22]), which was not the scenario in the present case. It is important to note that the micro-mechanical properties of the bulk Inconel 718 alloy reported in this work were obtained via micro-pillar compression, which may differ from the reported values in the literature, which was obtained through traditional macro-scale testing, mostly tensile in nature.

**Figure 7:**
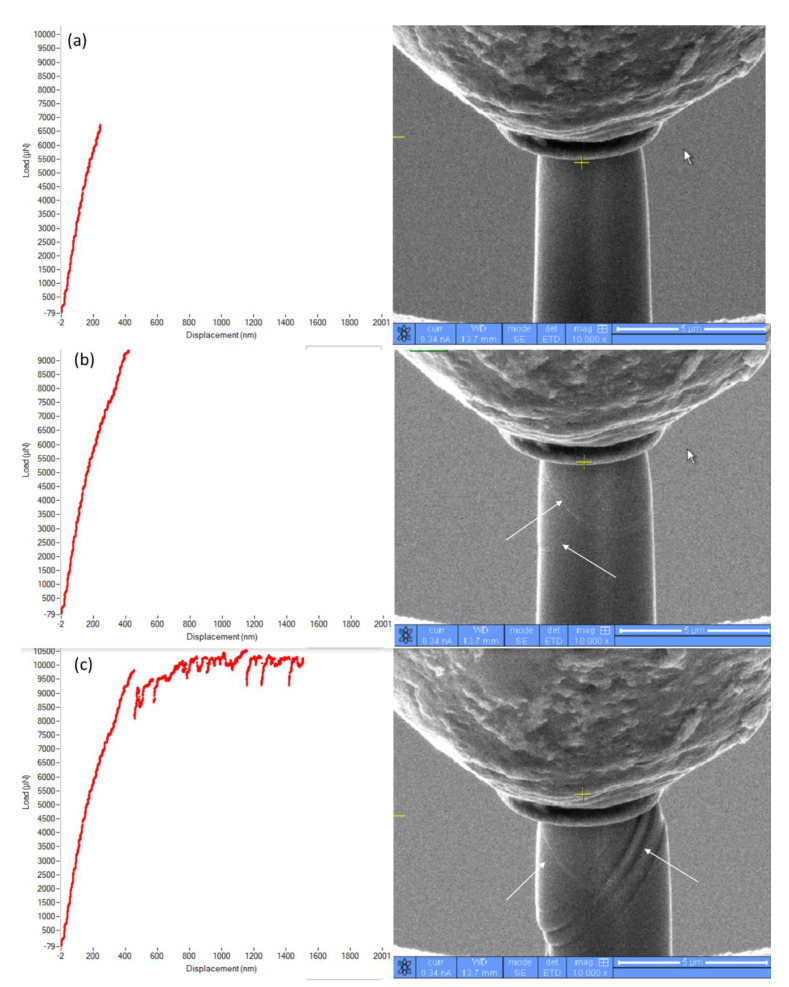
State of the micro-pillar at different strain intervals in the course of compression: (**a**) 1%, (**b**) 3%, and (**c**) 10% strain.

These unique features that appear in stress–strain graphs during compression can be correlated with the stages of physical deformation in the course of compression, as shown in Figure 7. The micrographs of Figure 7 were derived from the video that was recorded during compression. At the beginning of compression, there was no visible physical deformation (Figure 7a), and mostly elastic deformation took place until the formation and propagation of the slip/shear planes (Figure 7b) took over the deformation mechanism. These continue with a drop in stress due to the propagation of the slip/shear plane further (Figure 7c) till complete fracture occurs along the width of the micro-pillar. A representative video of the whole compression process was provided as  Supplementary Material.

## 4. Conclusions

Microstructural characterization and micro-mechanical properties of the recast layer that took place in the course of WEDM of Inconel 718 alloy have been investigated in this study. The machined surface shows the presence of re-solidified materials, depressions, and shallow cracks, which need to be removed prior to any application. The thickness of the recast layer was about 6.2 ± 2.1 µm, without the presence of a heat-affected zone. TEM results confirm the formation of metallurgical bonding between the interface, with a change in the direction of lattice orientation, together with the presence of various particles in the recast layer. The superficial layer exhibits much higher yield strength (762.7 ± 11.1 MPa), ultimate compressive strength (1151.6 ± 51.2 MPa), and Young’s modulus (406.6 ± 23.6 MPa) compared to the bulk material.

## Figures and Tables

**Table 1:** Micro-mechanical properties of the recast layer and bulk material.

Material	Superficial Layer	Bulk Material
**Yield strength (σ_y_), MPa**	762.7 ± 11.1	360.9 ± 41.8
**Ultimate compressive strength (σ_UTS_), MPa**	1151.6 ± 51.1	523.2 ±22.1
**Young’s modulus (E), MPa**	406.6 ± 23.5	222.6 ± 49.2

## Data Availability

The raw/processed data used to produce the results will be made available by the corresponding author upon reasonable request.

## Supplementary Materials

The complete video of in-situ micropillar compression of the recast layer together with corresponding load-displacement curve could be found from the following link: https://universityofadelaide.box.com/s/48sfjgc2ts3exo8gdlyme3rsa6ithueh (accessed on 15 January 2023).
